# Nicotine Affects Bone Resorption and Suppresses the Expression of Cathepsin K, MMP-9 and Vacuolar-Type H^+^-ATPase d2 and Actin Organization in Osteoclasts

**DOI:** 10.1371/journal.pone.0059402

**Published:** 2013-03-15

**Authors:** Hideki Tanaka, Natsuko Tanabe, Takayuki Kawato, Kumiko Nakai, Taro Kariya, Sakurako Matsumoto, Ning Zhao, Masafumi Motohashi, Masao Maeno

**Affiliations:** 1 Department of Oral Health Sciences, Nihon University School of Dentistry, Tokyo, Japan; 2 Department of Biochemistry, Nihon University School of Dentistry, Tokyo, Japan; 3 Division of Functional Morphology, Dental Research Center, Nihon University School of Dentistry, Tokyo, Japan; 4 Nihon University Graduate School of Dentistry, Tokyo, Japan; 5 Department of Endodontics, School of Dentistry, Shandong University, Jinan, Shandong Province, People's Republic of China; Faculté de médecine de Nantes, France

## Abstract

Tobacco smoking is an important risk factor for the development of several cancers, osteoporosis, and inflammatory diseases such as periodontitis. Nicotine is one of the major components of tobacco. In previous study, we showed that nicotine inhibits mineralized nodule formation by osteoblasts, and the culture medium from osteoblasts containing nicotine and lipopolysaccharide increases osteoclast differentiation. However, the direct effect of nicotine on the differentiation and function of osteoclasts is poorly understood. Thus, we examined the direct effects of nicotine on the expression of nicotine receptors and bone resorption-related enzymes, mineral resorption, actin organization, and bone resorption using RAW264.7 cells and bone marrow cells as osteoclast precursors. Cells were cultured with 10^−5^, 10^−4^, or 10^−3^ M nicotine and/or 50 µM α-bungarotoxin (btx), an 7 nicotine receptor antagonist, in differentiation medium containing the soluble RANKL for up 7 days. 1–5, 7, 9, and 10 nicotine receptors were expressed on RAW264.7 cells. The expression of 7 nicotine receptor was increased by the addition of nicotine. Nicotine suppressed the number of tartrate-resistant acid phosphatase positive multinuclear osteoclasts with large nuclei(≥10 nuclei), and decreased the planar area of each cell. Nicotine decreased expression of cathepsin K, MMP-9, and V-ATPase d2. Btx inhibited nicotine effects. Nicotine increased CA II expression although decreased the expression of V-ATPase d2 and the distribution of F-actin. Nicotine suppressed the planar area of resorption pit by osteoclasts, but did not affect mineral resorption. These results suggest that nicotine increased the number of osteoclasts with small nuclei, but suppressed the number of osteoclasts with large nuclei. Moreover, nicotine reduced the planar area of resorption pit by suppressing the number of osteoclasts with large nuclei, V-ATPase d2, cathepsin K and MMP-9 expression and actin organization.

## Introduction

Tobacco smoking, which is strongly associated with an increased risk of cancer [1] and cardiovascular disease [2], [3], has also been implicated as a risk factor in postmenopausal osteoporosis with effects on bone content and the risk of fracture [4], [5]. These risks are partly tobacco-associated impairment of normal immunological surveillance and defense mechanisms, such as neutrophil and macrophage function [6] and/or the cellular immune response [7]. In this regard, tobacco smoking contributes to the progress of chronic inflammatory periodontal disease.

Tobacco contains a complex mixture of substances, including nicotine, various nitrosamines, trace elements, and various poorly characterized substances. Many of the undesirable effects of tobacco have been attributed to nicotine, a major component of the particulate phase of tobacco smoke. Nicotine induces vascular changes in gingival tissue [8], [9] which are similar to the exudative vasculitis that is characteristic of the initial lesion in periodontal inflammation [10]–[12]. Several studies have examined the *in vitro* effects of nicotine on the function of epithelial cells, fibroblasts, and osteoblasts [11], [12]. These results indicated that nicotine itself may augment the destruction of the gingival extracellular matrix that occurs during periodontal inflammation that is associated with smokeless tobacco use.

Osteoclasts are large multinucleated cells with the unique capability of extracellular resorption of the mineralized matrices of bone, teeth, and mineralized cartilage. The actions of osteoclasts and osteoblasts are vital for skeletal development and remodeling. The balance between resorption and formation is critical for skeletal homeostasis, and an imbalance leads to diseases such as osteoporosis [13], [14]. Inflammatory diseases such as periodontitis also cause a local imbalance in resorption and formation. Bone resorption occurs under the aegis of a cytoskeletal structure in the osteoclast known as the ruffled border. The ruffled border forms by polarization of cytoplasmic vesicles to the bone-apposed plasma membrane into which they are inserted, leading to the enhanced complexity of osteoclasts. Mature osteoclasts secrete hydrogen ions (H^+^), which are produced via carbonic anhydrase II (CA II), from this ruffled border. By this mechanism, the vesicles deliver an electrogenic vacuolar-type H^+^-ATPase (V-ATPase) or proton pump and a chloride channel into the ruffled border, thus acidifying the resorptive space. Mature osteoclasts also secrete proteinases such as cathepsin K and matrix metalloproteinase (MMP)-9, which are needed to degrade the organic matrix of bone in the microenvironment of low pH surrounded by actin filaments [15]. Both V-ATPase and actin filaments interact with osteoclasts *in vivo* [16].

Our previous studies have indicated that nicotine suppresses mineralized nodule formation [17] and induces the expression of MMPs in osteoblasts [18]. Additionally, we reported that nicotine and lipopolysaccharide enhance osteoclast differentiation through macrophage colony-stimulating factor and prostaglandin E_2_ production, which are induced by nicotine-treated osteoblasts [19], and stimulate the resorption process that occurs during turnover of osteoid by osteoblasts [20]. However, the direct effects of nicotine on the bone resorption mechanism by osteoclasts are unknown. Thus, we examined the direct effect of nicotine on osteoclast differentiation and the expression of nicotine receptors and bone resorption-related enzymes such as CA II, cathepsin K, MMP-9, and V-ATPase in the presence or absence of α-bungarotoxins (btx), an α7 nicotine receptor antagonist, in RAW264.7 cells and mouse bone marrow cells as osteoclast precursors. Moreover, we examined the effect of nicotine on mineral resorption, bone resorption activity, and actin organization by nicotine-induced osteoclasts.

## Results

### Nicotine receptor expression

**Figure 1:**
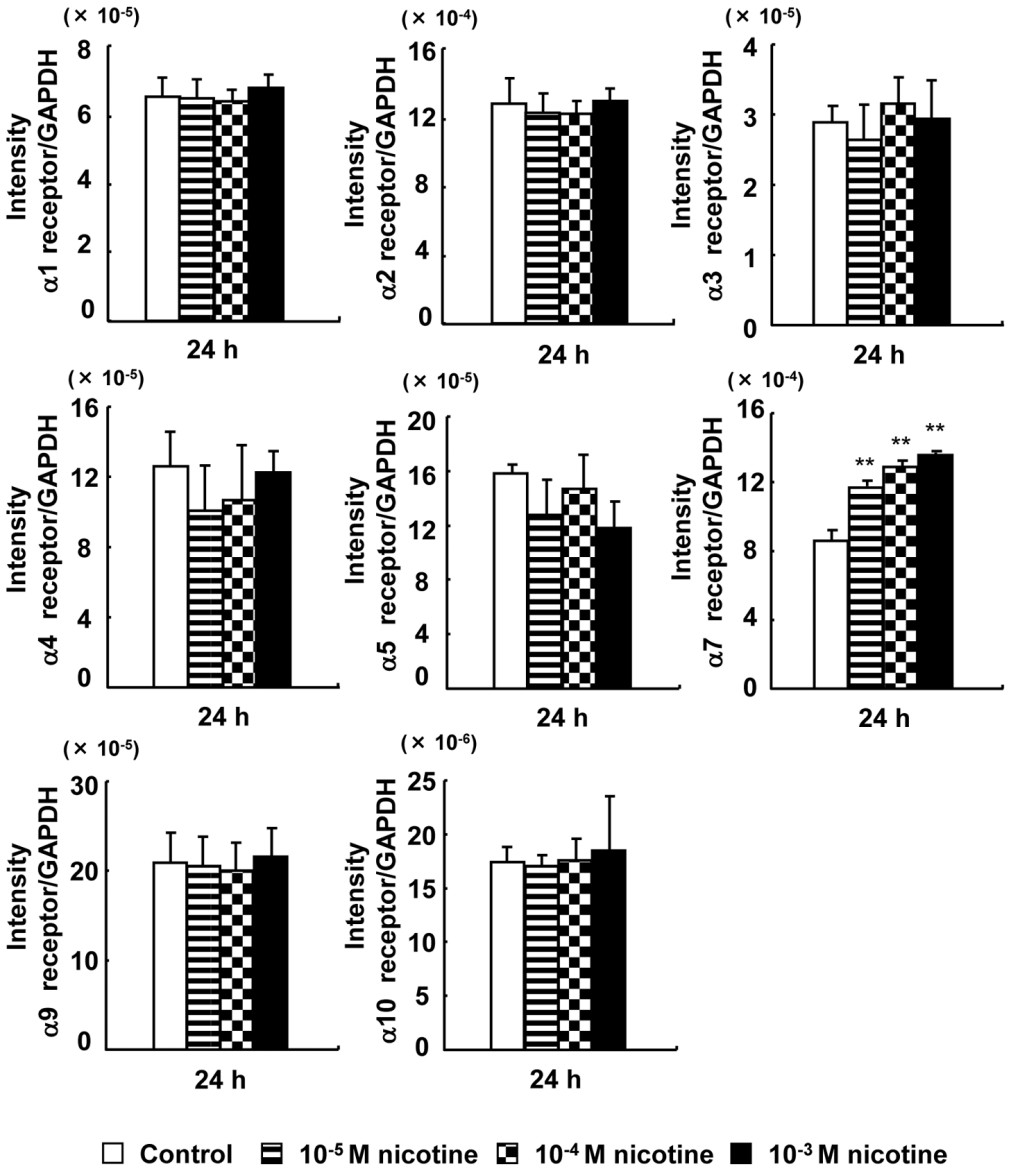
Effect of nicotine on the mRNA expression of nicotine receptors. RAW264.7 cells were cultured in differentiation medium with 0 (control), 10^−5^, 10^−4^, or 10^−3^ M nicotine for 24 h, and the expression of nicotine receptors (α1–7, α9, and α10) at the mRNA level was determined with real-time PCR. Data are expressed as the mean±standard deviation (S.D.), n = 3 independent experiments. *p<0.05, **p<0.01, nicotine treatment vs. control.

The 1–5, 7, 9, and 10 nicotine receptors were expressed in RAW264.7 cells. The α6 nicotine receptor was not detected (Fig. 1). The expression of 7 nicotine receptor was significantly increased by nicotine in a dose-dependent manner.

### TRAP staining of osteoclasts and spreading in osteoclasts

**Figure 2:**
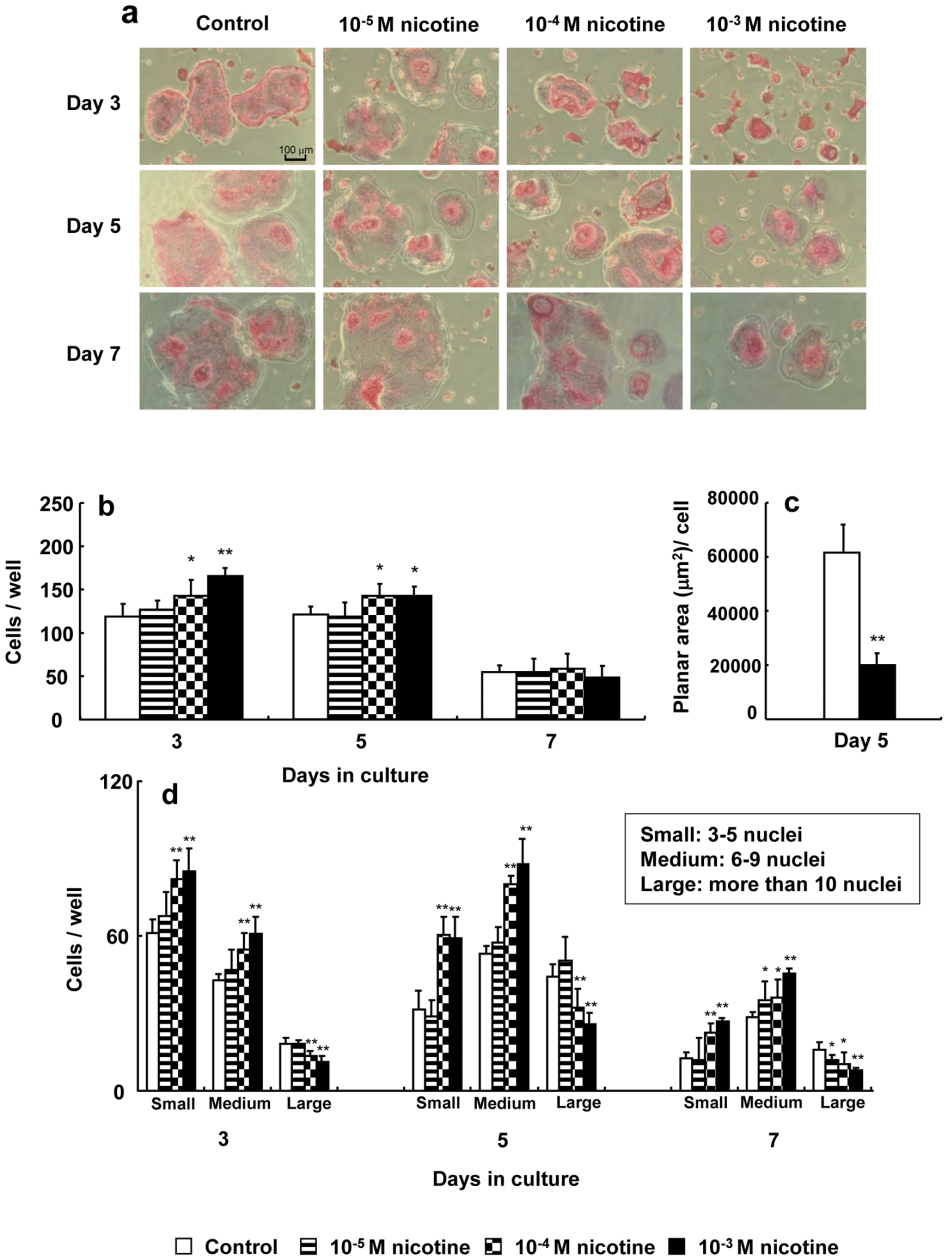
Effect of nicotine on tartrate-resistant acid phosphatase (TRAP) staining of osteoclasts. RAW264.7 cells were cultured in differentiation medium with 0 (control), 10^−5^, 10^−4^, or 10^−3^ M nicotine for up to 7 days, and then were stained using a TRAP staining kit on days 3, 5, and 7 of culture (original magnification, ×100) (a). TRAP-positive cells with more than three nuclei were counted under a phase-contrast microscope on days 3, 5, and 7 of culture. Data are shown as the mean±S.D., n = 3 independent experiments, each performed using quintuplicate wells. *p<0.05, **p<0.01, nicotine treatment vs. control (b). The cells were quantified by tracing the perimeter of each osteoclast to obtain the mean planar cell area under each condition on day 5 of culture. The histogram shows the average planar area of osteoclasts under the control or 10^−3^ M nicotine condition. Data are expressed as the mean±S.D., n = 3 independent experiments, each performed using quintuplicate wells with five randomly selected osteoclasts analyzed per well. **p<0.01 compared with control (c). TRAP-positive cells with small (3–4 nuclei), medium (5–9 nuclei) and large (greater than 10 nuclei) were counted under a phase-contrast microscope on days 3, 5, and 7 of culture. Data are shown as the mean±S.D., n = 3 independent experiments, each performed using quintuplicate wells. *p<0.05, **p<0.01, nicotine treatment vs. control (d).

No change was observed in TRAP staining of osteoclasts following the addition of nicotine. However, the area of each osteoclast was gradually reduced by nicotine in a dose-dependent manner (Fig. 2a). The number of TRAP-positive multinucleated osteoclasts significantly increased by 1.2- to 1.5-fold in cell culture with 10^−4^ and 10^−3^ M nicotine compared to the control on days 3 and 5 of culture, respectively (Fig. 2b). The planar area of nicotine-treated osteoclasts was significantly decreased by 0.3-fold in cell culture with 10^−3^ M nicotine compared to control osteoclasts (Fig. 2c). Furthermore 10^−4^ and 10^−3^ M nicotine significantly suppressed the number of osteoclasts with large nuclei (>10) after 3, 5, and 7 days in culture days (Fig. 2d).

### Expression of CA II, cathepsin K, MMP-9, and V-ATPase

**Figure 3:**
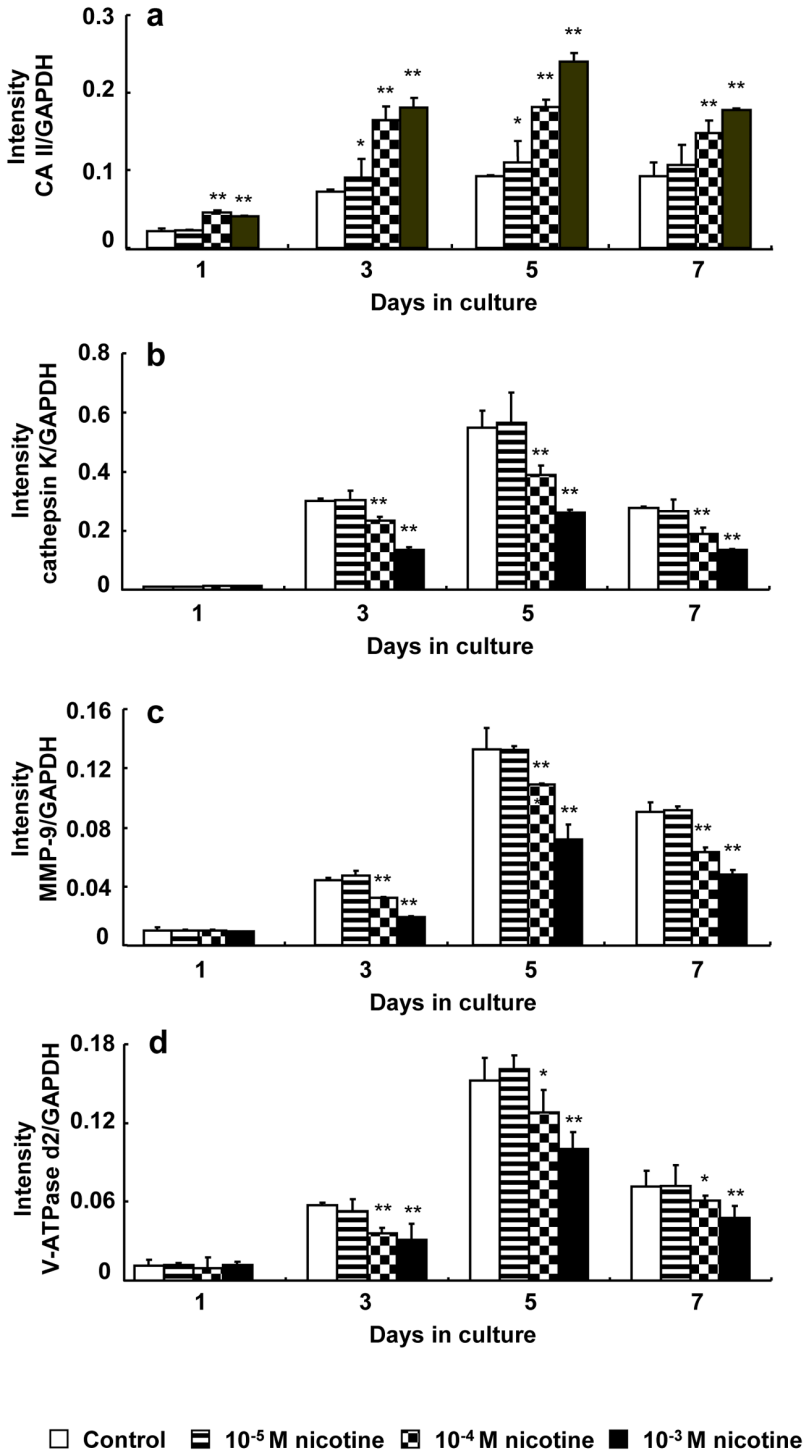
Effect of nicotine on mRNA expression of CA II, cathepsin K, MMP-9, and V-ATPase d2. RAW264.7 cells were cultured in differentiation medium with 0 (control), 10^−5^, 10^−4^, or 10^−3^ M nicotine for up to 7 days. The mRNA expression of CA II (a), cathepsin K (b), MMP-9 (c), and V-ATPase d2 (d) was determined using real-time PCR on days 1, 3, 5, and 7 of culture. Data are shown as the mean±S.D., n = 3 independent experiments. *p<0.05, **p<0.01, nicotine treatment vs. control.

Fig. 3 shows the effect of nicotine on the expression of CA II, cathepsin K, MMP-9, and V-ATPase d2 at the mRNA level. The expression gradually increased both in the presence and absence of nicotine until day 5 and then decreased on day 7 of culture. CA II expression increased in a dose-dependent manner after nicotine addition on days 3, 5, and 7 of culture. The expression significantly increased by 1.60- to 2.26 fold and 1.93- to 2.60-fold in cell culture with 10^−4^ and 10^−3^ M nicotine, respectively, compared to each control (Fig. 3a). In contrast, the expression of cathepsin K, MMP-9, and V-ATPase d2 decreased in a dose-dependent manner after nicotine addition on days 3, 5, and 7 of culture (Fig. 3b–d). Cathepsin K expression significantly decreased by 0.68- to 0.78-fold and 0.45- to 0.49-fold in cell culture with 10^−4^ and 10^−3^ M nicotine, respectively, compared to each control (Fig. 3b). MMP-9 expression significantly decreased by 0.72- to 0.82-fold and 0.43- to 0.54-fold in cell culture with 10^−4^ and 10^−3^ M nicotine, respectively, compared to each control (Fig. 3c). V-ATPase d2 expression significantly decreased by 0.63- to 0.84-fold and 0.54- to 0.67-fold in cell culture with 10^−4^ and 10^−3^ M nicotine, respectively, compared to each control (Fig. 3d).

**Figure 4:**
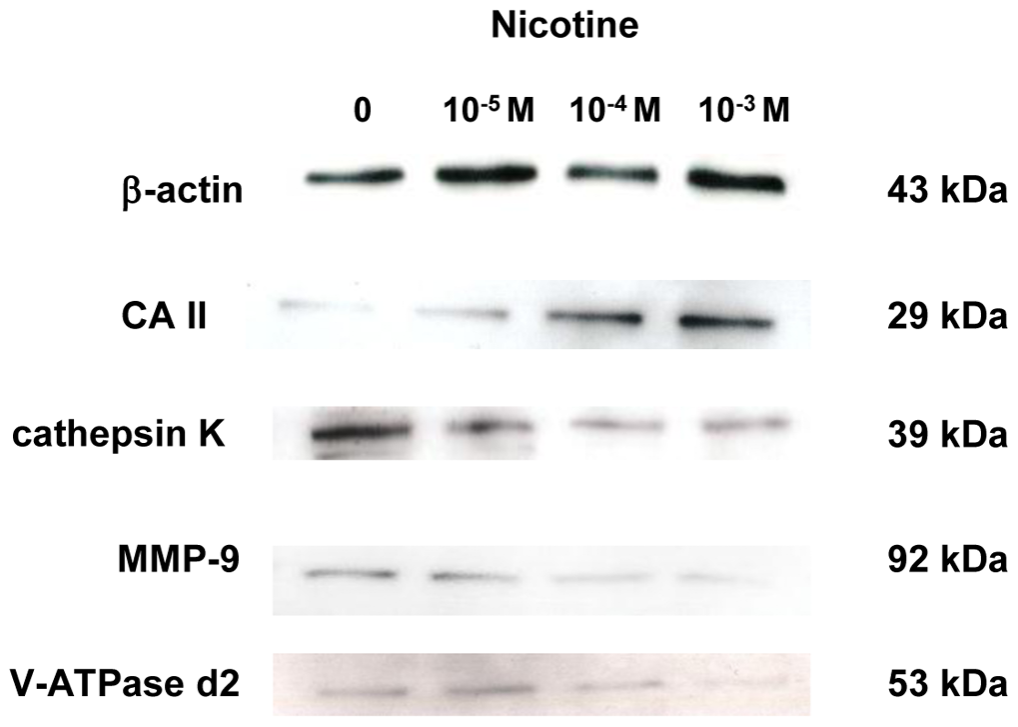
Effect of nicotine on protein expression of CA II, cathepsin K, MMP-9, and V-ATPase d2. RAW264.7 cells were cultured in differentiation medium with 0 (control), 10^−5^, 10^−4^, or 10^−3^ M nicotine for 5 days. The protein expression of CA II, cathepsin K, MMP-9, and V-ATPase was determined by Western blotting.

Fig. 4 shows the effect of nicotine on the expression of CA II, cathepsin K, MMP-9, and V-ATPase d2 at the protein level on day 5 of culture. CA II expression gradually increased in a dose-dependent manner after nicotine addition, whereas expression of cathepsin K, MMP-9, and V-ATPase d2 gradually decreased in a dose-dependent manner after nicotine addition.

**Figure 5:**
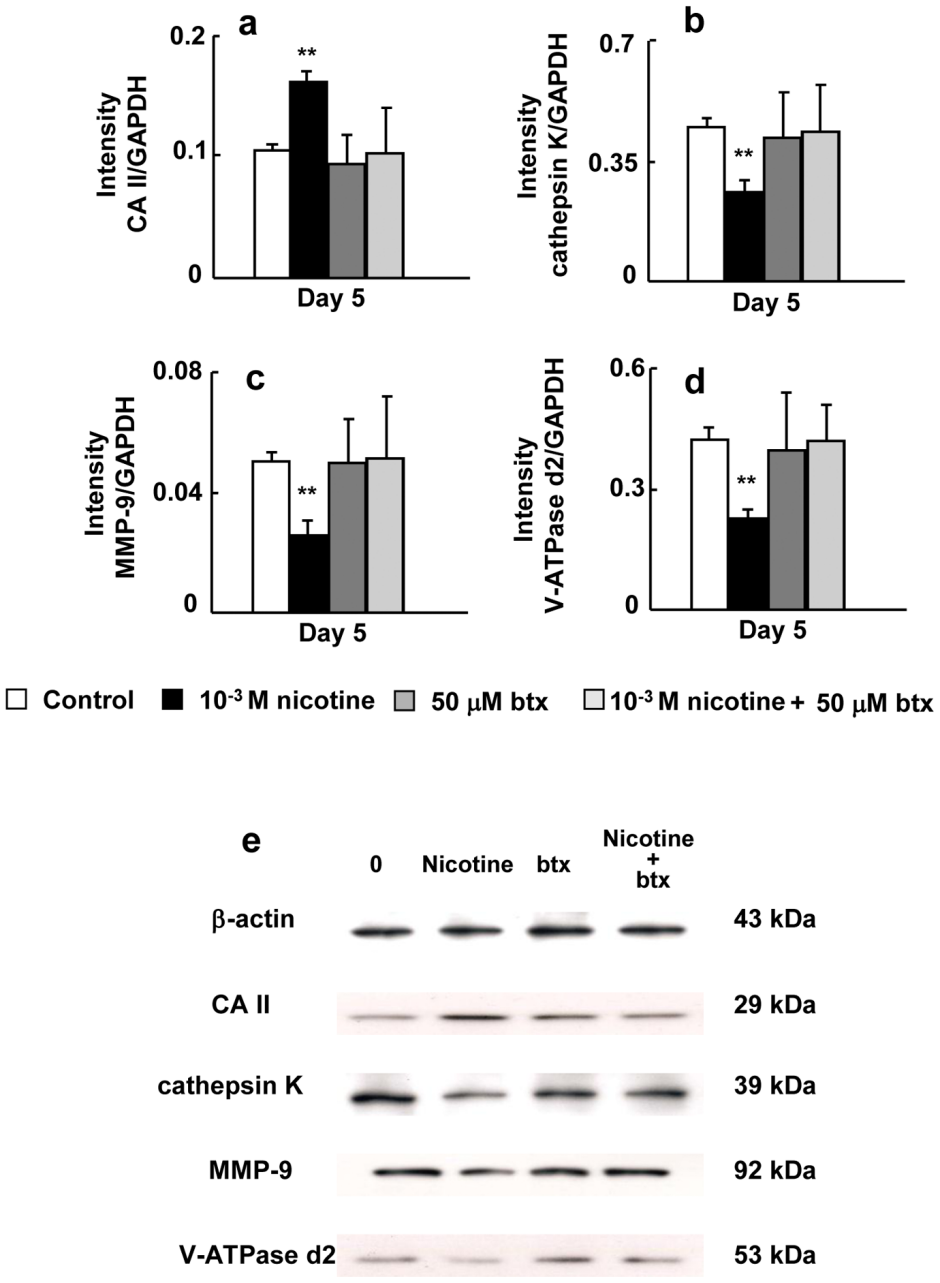
Effect of btx and/or nicotine on the expression of CA II, cathepsin K, and MMP-9 and V-ATPase d2. RAW264.7 cells were cultured in differentiation medium without drug (control), 50 µM btx, 10^−3^ M nicotine, or 50 µM btx and 10^−3^ M nicotine for 5 days. The mRNA expression of CA II (a), cathepsin K (b), MMP-9 (c), and V-ATPase d2 (d) was determined by real-time PCR. Cells were cultured in differentiation medium without drug (control), 50 µM btx, 10^−3^ M nicotine, or 50 µM btx and 10^−3^ M nicotine for 5 days. The protein expression of CA II, cathepsin K, MMP-9, and V-ATPase d2 was determined by Western blotting (e). Data are shown as the mean±S.D., n = 3 independent experiments. *p<0.05, **p<0.01, nicotine treatment vs. control.

Fig. 5 shows the effects of nicotine and/or btx on the expression of CA II, cathepsin K, MMP-9, and V-ATPase d2 at the mRNA level on day 5 of culture in RAW264.7 cells. When btx was present in the culture with nicotine, it blocked the inductive effect of nicotine on CA II expression (Fig. 5a) and the suppressive effect of nicotine on the expression of cathepsin K, MMP-9, and V-ATPase d2 (Fig. 5b–d). The level of CA II, cathepsin K, MMP-9, and V-ATPase d2 in cells treated with both btx and nicotine was similar to that in each control. Fig. 5e shows the effects of nicotine and/or btx on CA II, cathepsin K, MMP-9 and V-ATPase d2 at the protein level on day 5 of culture. Btx inhibited the effects of nicotine on CA II, cathepsin K, MMP-9 and V-ATPase d2 protein expression.

**Figure 6:**
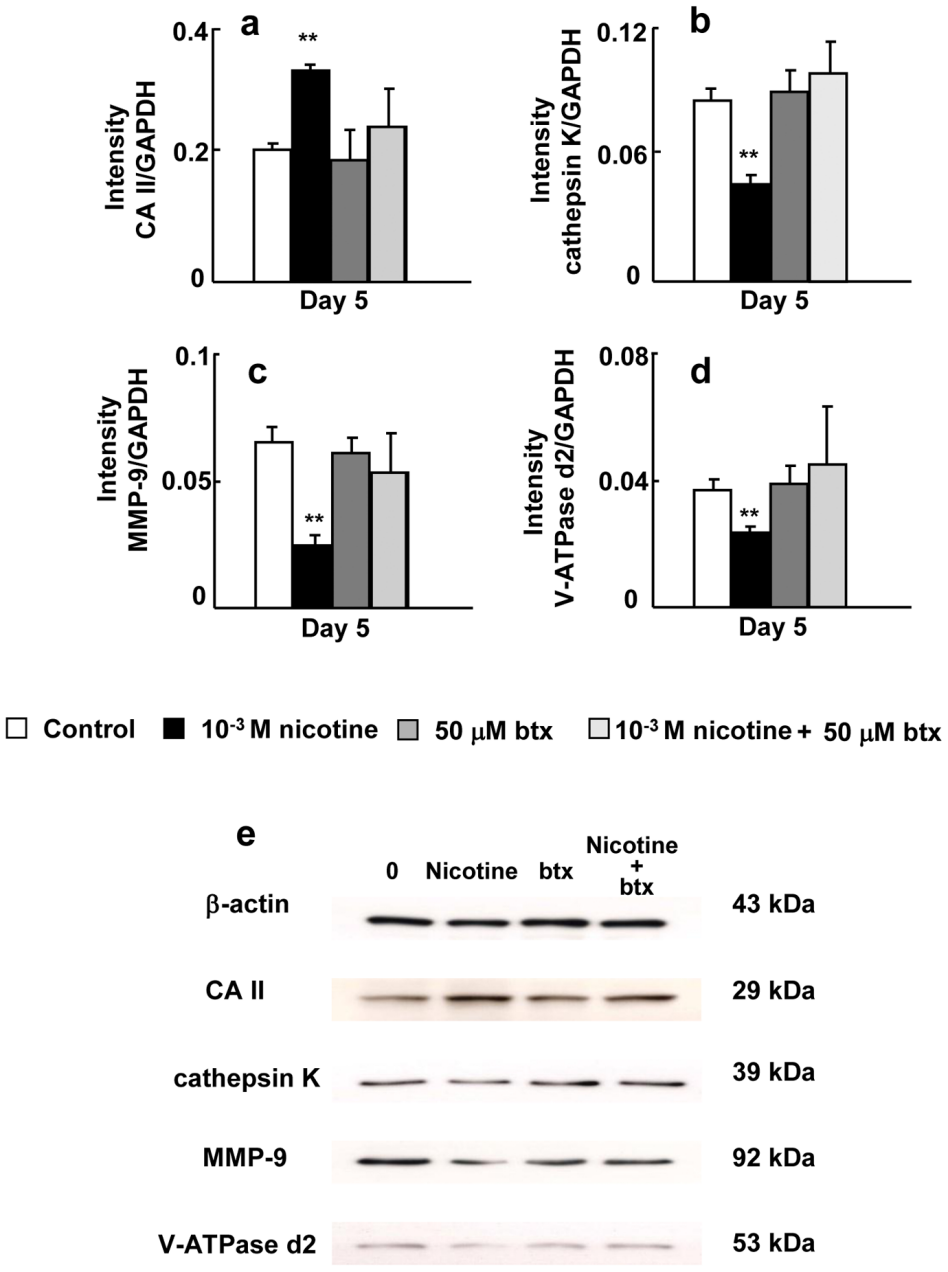
Effect of btx and/or nicotine on the expression of CA II, cathepsin K, and MMP-9 and V-ATPase d2. Bone marrow cells were cultured in medium containing macrophage colony-stimulating factor (50 ng/ml), and RANKL (50 ng/ml) without drug (control), 50 µM btx, 10^−3^ M nicotine, or 50 µM btx, and 10^−3^ M nicotine for 5 days. The mRNA expression of CA II (a), cathepsin K (b), MMP-9 (c), and V-ATPase d2 (d) was determined by real-time PCR. The protein expression of CA II, cathepsin K, MMP-9, and V-ATPase d2 was determined by Western blotting (e).Data are shown as the mean±S.D., n = 3 independent experiments. *p<0.05, **p<0.01, nicotine treatment vs. control.

Fig. 6 shows the effects of nicotine and/or btx on the expression of CA II, cathepsin K, MMP-9, and V-ATPase d2 at the mRNA level on day 5 of culture in bone marrow cells. When btx was present in the culture with nicotine, it blocked the inductive effect of nicotine on CA II expression (Fig. 6a) and the suppressive effects of nicotine on the expression of cathepsin K, MMP-9, and V-ATPase d2 (Fig. 6b–d). The level of the CA II, cathepsin K, MMP-9, and V-ATPase d2 in cells treated with both btx and nicotine was similar to that in each control. Fig. 6e shows the effects of btx on CA II, cathepsin K, MMP-9 and V-ATPase d2 at the protein level on day 5 of culture. Btx inhibited the effects of nicotine on CA II, cathepsin K, MMP-9 and V-ATPase d2 protein expression.

### Nicotine suppresses actin organization

**Figure 7:**
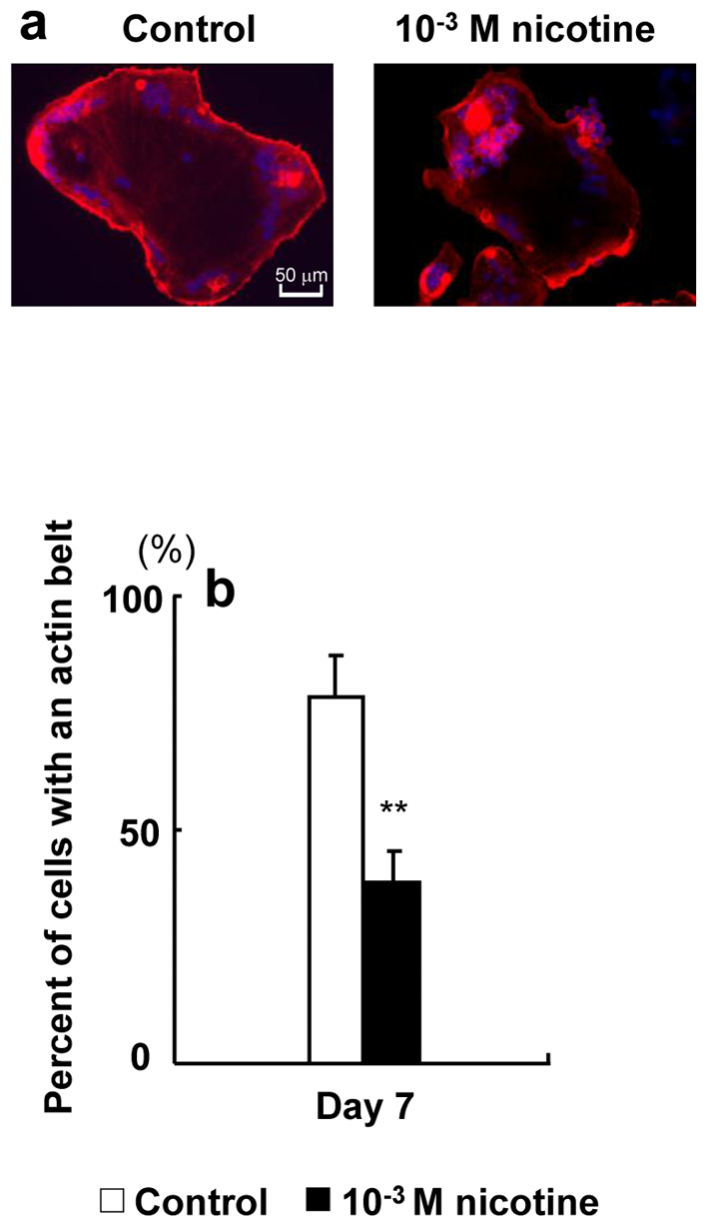
Effect of nicotine on actin organization. RAW264.7 cells were plated onto 12-mm coverslips. The cells were cultured with 0 (control) or 10^−3^ M nicotine in differentiation medium for 7 days and then fixed. Filamentous actin was labeled with fluorescently tagged phalloidin (red), nuclei were labeled with 4′,6-diamidino-2-phenylindole (DAPI, Vector Laboratories) (blue), and osteoclasts were observed by fluorescence microscopy. Representative micrograph of osteoclasts on a coverslip (original objective, ×40) (a). The percentage of osteoclasts exhibiting actin belts under each condition was quantified (b). Data are expressed as the mean±S.D., n = 3 independent experiments, each performed using triplicate coverslips with five randomly selected osteoclasts analyzed per coverslip. **p<0.01 nicotine treatment vs. control.

Fig. 7a shows an image of actin organization in osteoclasts after 7 days in culture. Osteoclasts exhibit small punctate F-actin-containing adhesion structures known as podosomes that often organize into a belt at the cell periphery. Nicotine inhibited actin belt formation compared to the control.

Fig. 7b shows the percentage of osteoclasts exhibiting the actin belt. Nicotine significantly suppressed the formation of an actin belt by 0.4-fold compared to the control.

### The effect of nicotine on mineral resorption activity

**Figure 8:**
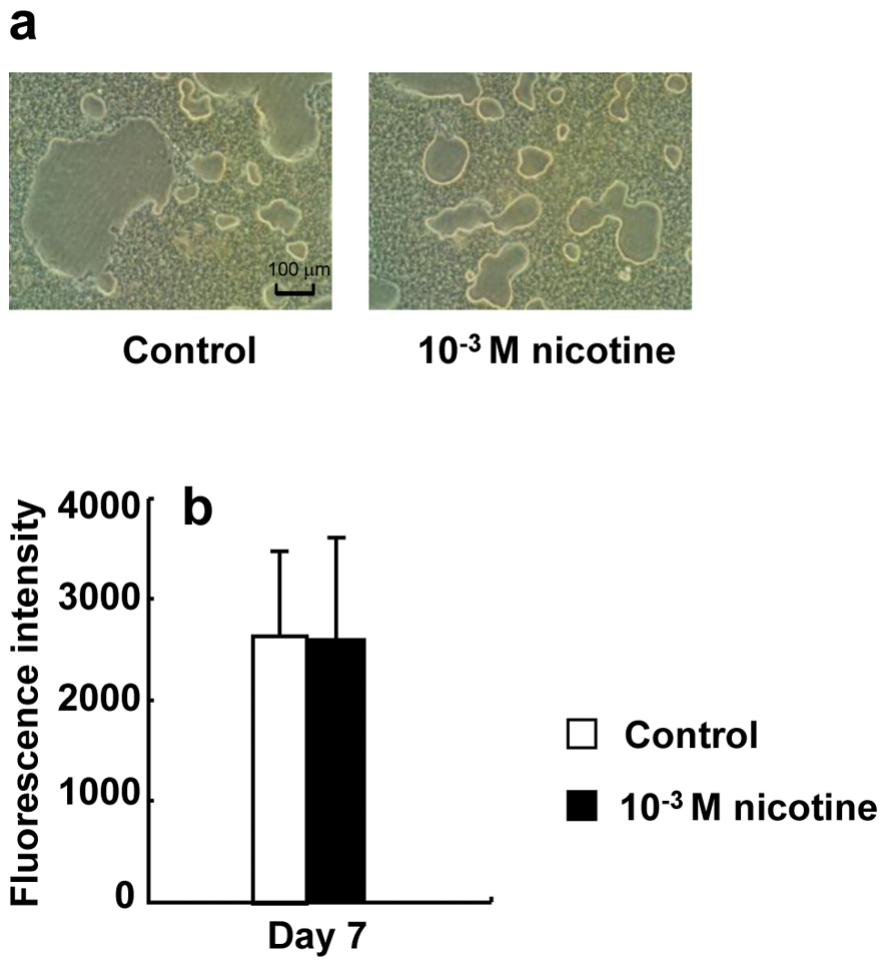
Effect of nicotine on mineral resorption activity. RAW264.7 cells were plated onto Bone Resorption Assay Kit 24 plates and cultured in differentiation medium with 0 (control) or 10^−3^ M nicotine for 7 days. Representative mineral resorption of control or 10^−3^ M nicotine by osteoclasts (a). Calcium phosphate released from the plate by activation of mineral resorption was quantified by fluorescence intensity using fluorescein isothiocyanate (FITC) (b). Data are expressed as the mean±S.D., n = 3 independent experiments, each performed using triplicate coverslips with five randomly selected osteoclasts analyzed per coverslip.

Pit formation was observed under all conditions (Fig. 8a). Nicotine did not affect the pit-forming area or calcium elution (Fig. 8b).

### The effect of nicotine on bone resorption activity

**Figure 9:**
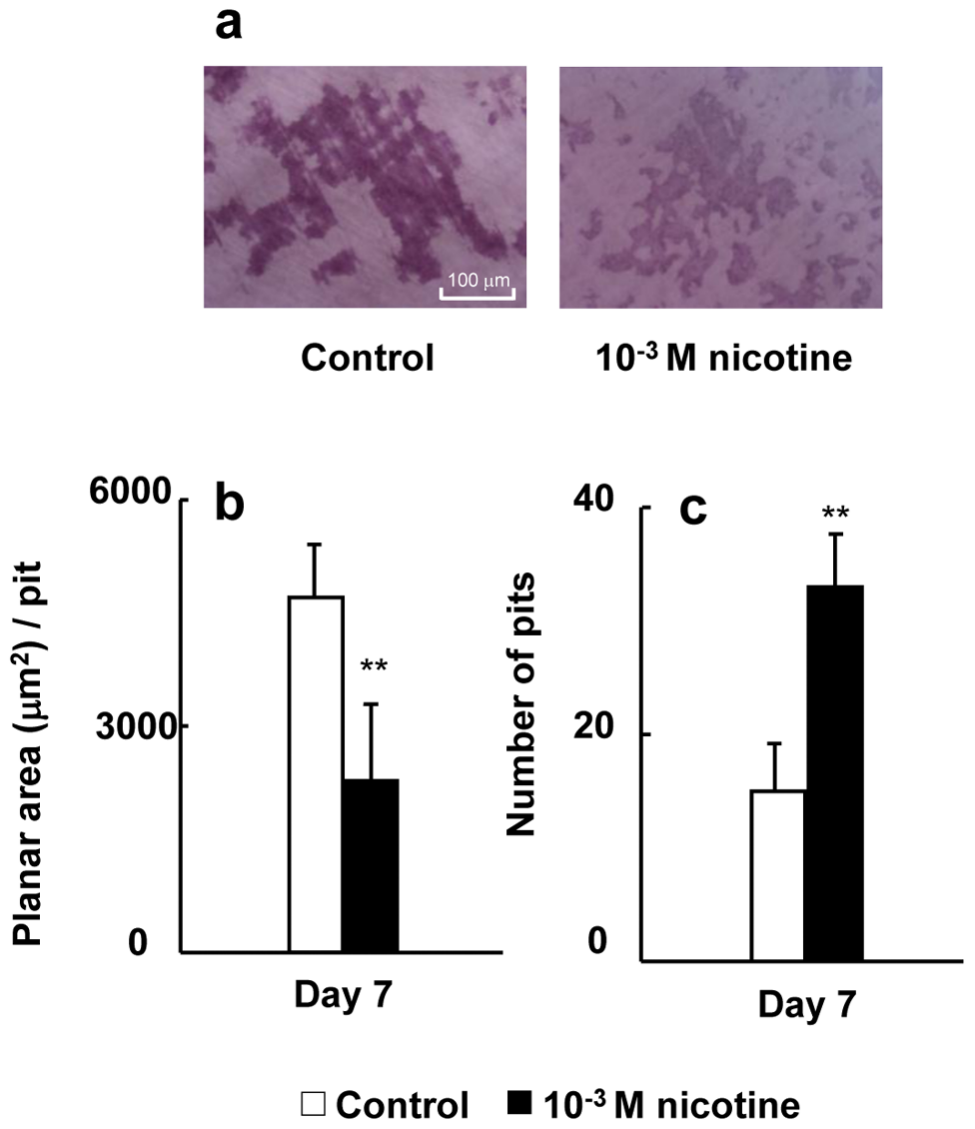
Effect of nicotine on pit formation. RAW264.7 cells were plated onto dentin slices and cultured in differentiation medium with 0 (control) or 10^−3^ M nicotine for 7 days. Representative the resorption pits of control or 10^−3^ M nicotine by osteoclasts (a). The pits were quantified by tracing the perimeter of each pit to obtain the mean planar pit area under each condition. The histogram shows the average planar area of each pit under the control or 10^−3^ M nicotine condition on day 7 of culture (b). Data are expressed as the mean±S.D., n = 3 independent experiments, each performed using triplicate coverslips with five randomly selected osteoclasts analyzed per coverslip The number of pits was counted under a phase-contrast microscope on day 7 of culture (c). Data are shown as the mean±S.D., n = 3 independent experiments, each performed using quintuplicate wells. **p<0.01, nicotine treatment vs. control. **p<0.01 nicotine treatment vs. control.

**Figure 10:**
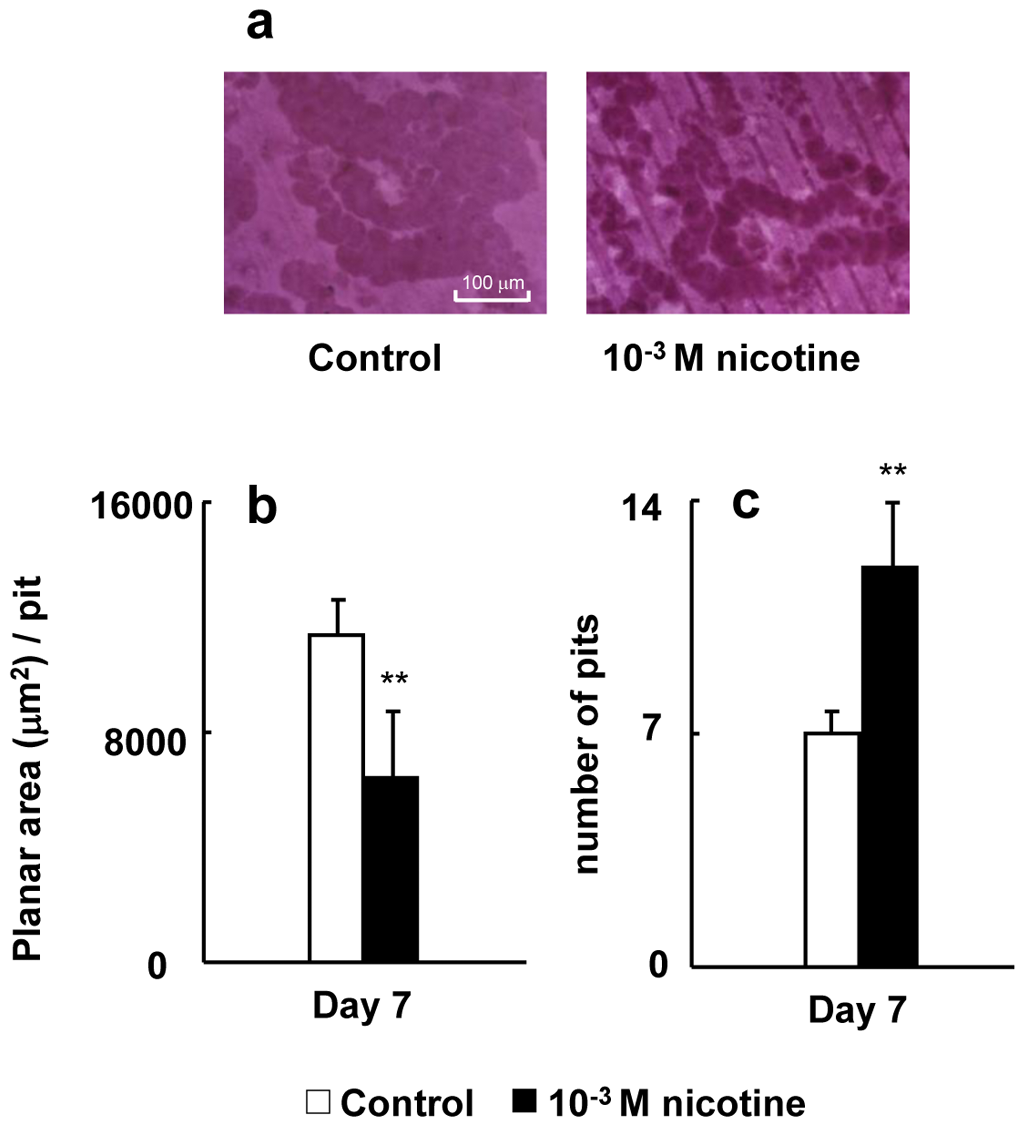
Effect of nicotine on pit formation. Bone marrow cells were plated onto dentin slices and cultured in medium containing macrophage colony-stimulating factor (50 ng/ml), and RANKL (50 ng/ml) with 0 (control) or 10^−3^ M nicotine for 7 days. Representative resorption pits of control or 10^−3^ M nicotine by osteoclasts (a). The pits were quantified by tracing the perimeter of each pit to obtain the mean planar pit area under each condition. The histogram shows the average planar area of each pit under the control or 10^−3^ M nicotine condition on day 7 of culture (b). Data are expressed as the mean±S.D., n = 3 independent experiments, each performed using triplicate coverslips with five randomly selected osteoclasts analyzed per coverslip. **p<0.01 nicotine treatment vs. control. The number of pits was counted under a phase-contrast microscope on day 7 of culture (c). Data are shown as the mean±S.D., n = 3 independent experiments, each performed using quintuplicate wells. **p<0.01, nicotine treatment vs. control.

Pit formation on a dentin slice was observed under all conditions (Figs. 9a, RAW264.7 cells; and 10a, bone marrow-derived osteoclasts). Nicotine suppressed the planar area per pit, whereas it enhanced the number of pits in both RAW264.7 cells (Figs. 9b and 9c) and bone marrow-derived osteoclasts (Figs. 10b and 10c).

## Discussion

We conducted the present study to clarify the direct effects of nicotine on the differentiation of osteoclast precursors and the function of mature osteoclasts. We used RAW264.7 cells and mouse bone marrow cells as osteoclast precursors. Our previous study indicated that RAW264.7 cells differentiate into osteoclasts in the presence of 50 ng/ml receptor activator of NF-κB ligand (RANKL), and the expression of CA II, cathepsin K, and MMP-9 in RAW264.7 cells is induced by the RANKL [21]. Thus, in the present study, we used a differentiation medium of DMEM containing 50 ng/ml RANKL to culture for RAW264.7 cells.

Several studies have indicated that tobacco smoking is an important risk factor for the development and severity of periodontitis [22]–[24]. To select the experimental concentration of nicotine, we assumed that nicotine infiltrates the gingival epithelium and influences the function of osteoclast precursors in alveolar bone. Ryder et al. [25] reported that acute smoke exposure resulted in elevation of nicotine in gingival crevicular fluid to 5961 ng/ml after smoking. We previously showed that 10^−4^ and/or 10^−3^ M nicotine inhibits mineralized nodule formation and the production of extracellular matrix proteins such as type I collagen and osteopontin by osteoblasts [17]. In addition, 10^−3^ M nicotine and lipopolysaccharide stimulate the formation of osteoclast-like cells by increasing macrophage colony-stimulating factor and prostaglandin E_2_ production by osteoblasts [19]. Thus, we used a concentration of 10^−5^ to 10^−3^ M nicotine, which is considerably higher than the nicotine level in serum.

In the present study, we observed that 1–5, 7, 9, and 10 nicotine receptors were expressed on RAW264.7 cells, and that expression of the 7 nicotine receptor increased in a dose-dependent manner with nicotine treatment. Additionally, the specific α7 nicotine receptor antagonist btx blocked the inductive effects of nicotine on CA II expression and the suppressive effects of nicotine on the expression of cathepsin K, MMP-9, and V-ATPase d2 in RAW264.7 cells and bone marrow-derived osteoclasts. Li et al. [26] reported that interleukin-10 and lipopolysaccharide increase the 7 nicotine receptor expression in RAW264.7 cells. Nicotine from smoking enhances α7 nicotine receptor expression on blood monocytes, and this may contribute to cholinergic immunomodulation. Furthermore, α7 nicotine receptor was only detectable in isolated blood monocytes of smokers [27]. Therefore a possible mechanism, consistent with our results is nicotine may mainly bind to the α7 receptors of both of cell types, and may also affect the expression of CA II, cathepsin K, MMP-9 and V-ATPase d2 via α7 receptors.

Our previous study reported that conditioned medium from nicotine-and/or lipopolysaccharide-treated osteoblasts increases the number of osteoclasts and TRAP-positive multinucleated osteoclast-like cells compared to untreated osteoblasts. However, we indicated that nicotine suppressed the planar area of osteoclast and the number of nuclei per osteoclast, whereas nicotine increased the number of osteoclasts in the present study. Furthermore we also showed that nicotine significantly suppressed the expression of V-ATPase d2 compared to control. Lee et al. [28] reported that V-ATPase d2 deficient mice have osteoclasts with reduced surface area, reduced the number of multinucleated TRAP positive cells and slightly increased number of mononuclear TRAP positive cells compared to wild-type mice. Thus, these results suggest that direct stimulation with nicotine induced the number of osteoclasts, whereas nicotine suppressed the fusion of nuclei in osteoclasts by suppressing V-ATPase d2 expression.

We examined the effect of nicotine on the expression of bone resorption-related enzymes, such as cathepsin K, MMP-9, and V-ATPase, at the mRNA and protein levels. Previously, David et al. reported that CA II is a target gene of c-fos/AP-1 in osteoclasts [29]. However, gene expression of MMP-9 and cathepsin K is regulated by TRAF6/NF-κB signaling pathway [30], [31]. CA II catalyzes the conversion of H_2_O and CO_2_ into H_2_CO_3_, a process that also occurs spontaneously, albeit at a low rate. H_2_CO_3_ then dissociates into H^+^ and HCO_3_^−^, and H^+^ is essential for the ability of osteoclasts to dissolve calcified bone matrix [32]. CA II and V-ATPase are involved in the extracellular acidification caused by osteoclasts. CA II generates H^+^ and HCO_3_^−^ via hydration of CO_2_, and the H^+^ ions are transported through the apical ruffled border of the osteoclasts to the resorption zone by V-ATPase [33]. The result is secretion of HCl into the resorptive microenvironment, producing a pH of ∼approximately 4.5[15]. However, Margolis et al. [34] reported that CA II-deficient mice only show a modest bone phenotype. Thus, CA II may not mainly affect bone resorption in nonacidic conditions.

The acidic milieu first mobilizes bone mineral; subsequently, the demineralized organic component of bone is degraded by the lysosomal proteinases cathepsin K and MMP-9 [33], [35]. Cathepsin K and MMP-9 are efficient collagenases that cleave both collagen types I and II [36]. In the present study, nicotine increased CA II expression, whereas it decreased the expression of V-ATPase d2 and lysosomal proteinases such as cathepsin K and MMP-9. Additionally, btx blocked the inductive effect of nicotine on CA II expression and the suppressive effect of nicotine on expression of cathepsin K, MMP-9, and V-ATPase d2. These results suggest that nicotine may have a negative effect on the RANKL-induced TRAF6/NF-κB signaling pathway, which affects cathepsin K and MMP-9 expression. However nicotine stimulates the c-fos/AP-1 signaling pathway which is involved in CA II gene expression. Nicotine also induces H^+^ production by increasing CA II expression, but the transportation of H^+^ through the apical ruffled border is suppressed by decreasing V-ATPase d2 expression.

We next examined the effect of nicotine on actin organization and the mineral resorption capacity in RAW264.7 cells. Characteristic organization of F-actin into a belt or ring-like structure with a double circle of vinculin around it is needed for formation of the sealing zone. This type of microfilament organization is typical only for resorbing osteoclasts and can thus be used as a marker for resorbing cells. These characteristic changes in the molecular organization of the cytoskeleton in osteoclasts during the resorption cycle offer several potential targets to inhibit bone resorption, that may be cell specific [37]. V-ATPase binds to actin filaments in osteoclasts and is immunocytochemically localized to the cell-bone attachment site. The interaction between V-ATPase and actin filaments is regulated by stimuli that activate osteoclast bone resorption [38]. Immunoelectron microscopy has shown that V-ATPase is present in the ruffled membrane, which is the resorptive organelle of the cell [15]. Proton secretion via V-ATPase represents a major process for regulating the systemic acid/base status, sperm maturation, and bone resorption. In the current study, nicotine markedly suppressed formation of the actin belt. This phenomenon may be involved with the change in cell shape such that the planar area of each osteoclast was reduced in a dose-dependent manner after nicotine addition in the present study. We plan to clarify the relevance of this finding in the future. We showed that nicotine did not affect the mineral resorption area and calcium elution. As mentioned above, nicotine induced H^+^ production by increasing CA II expression; conversely, nicotine inhibited the expression of V-ATPase, which secretes H^+^ from the ruffled border. The closed space of low pH that is surrounded by actin filaments is required to dissolve bone mineral by H^+^ [34]. Pennypacker et al. reported that young adult cathepsin K (^−^/^−^) mice have higher bone mass and thicker cortices than wild-type mice in both cortical and trabecular regions. This increase in cortical bone quantity is associated with decreased resorption and increased osteoclast numbers at surfaces adjacent to bone marrow. Cortical bone is stronger in cathepsin K (^−^/^−^) mice, commensurate with its increased bone mineral density and thicker cortices [39]. The most compelling evidence of MMP-9 that these enzymes participate in the resorptive process comes from the demonstration that bone resorption is attenuated in mice carrying a mutation in the site in type I collagen that is targeted by neutral collagenases [33]. ATP6ap1, an accessory subunit of V-ATPases [40], knockdown osteoclasts exhibited impaired lysosomal trafficking and exocytosis, as indicated by the absence of lysosomal trafficking to the ruffled border and a lack of cathepsin K exocytosis into the resorption lacuna [41]. Chung et al. also reported that V-ATPase blockade inhibited functional migration and invasion in pancreatic ductal adenocarcinoma cells with predominantly MMP-9 activity [42]. Thus, we confirmed the effect of nicotine on the bone resorption, and determined the pit-formation activity on dentin slices following nicotine stimulation in RAW264.7 cells and bone marrow-derived osteoclasts. Nicotine suppressed the planar area per pit although enhanced the number of pits in both cell type. These results suggest that nicotine reduces bone resorption by suppressing V-ATPase d2, cathepsin K and MMP-9 expression and actin organization. Previous studies reported that Benzo[a]pyrene (BaP), an environmental pollutant present in high concentrations in urban smog and cigarette smoke, inhibits osteoclast differentiation and bone resorption. BaP-mediated inhibition of osteoclastogenesis is a consequence of crosstalk between acetylcholine receptor (AhR) and RANKL signaling pathways competing for the common transcription factor NF-κB [43], [44].

*In vivo*, nicotine significantly decreases the trabecular bone volume, trabecular thickness, mineralizing surface, mineral appositional rate, and bone formation rate, while causing an increase in osteoclast surface. Moreover, nicotine also exerts negative effects on dynamic trabecular histomorphometric parameters [45]. Furthermore, we previously showed that nicotine suppresses mineralized nodule formation by osteoblasts *in vitro* [17]. These reports suggest that nicotine suppresses bone formation by osteoblasts. Conversely, we demonstrated here that nicotine reduces the planar area of the resorption pit by osteoclasts, and does not affect mineral resorption. During the process of bone remodeling, the balance between resorption and formation is important for skeletal homeostasis, and an imbalance leads to various bone diseases [12], [13]. Thus, we conclude that smokers who are exposed to nicotine for a long period of time may develop bone diseases such as alveolar bone loss with accompanying periodontitis, or osteoporosis which occurs due to an imbalance in bone remodeling in association with the suppressed function of osteoblasts and osteoclasts.

In conclusion, our results suggest that direct stimulation of osteoclast precursors by nicotine induces osteoclasts with the small number of nuclei and increased H^+^ production by increasing CA II expression in mature osteoclasts. However, nicotine reduced the number of osteoclasts with many nuclei and the planar area of the resorption pit by suppressing V-ATPase d2, cathepsin K and MMP-9 and actin organization. Therefore, *in vivo*, nicotine may act by suppressing the planar area of the resorption pit, expression of V-ATPase d2, cathepsin K and MMP-9 expression and actin organization, which finally results in decreased bone resorption. Taken together, our results indicate that nicotine may induce an imbalance of osteoblasts and osteoclasts *in vitro*, a phenomenon that further explains the stimulatory effects of nicotine on bone turnover *in vivo*.

## Materials and Methods

### Materials

Nicotine, Dulbecco's modified Eagle medium (DMEM), α-minimum essential medium (–MEM), α-bungarotoxin (btx) and dentin slices were purchased from Wako Fine Chemicals (Osaka, Japan). Soluble receptor activator of NF-κB ligand (RANKL) was obtained from R&D Systems (Minneapolis, MN, USA). Recombinant human macrophage colony-stimulating factor (Leukoprol) was obtained from Kyowa Hakko (Tokyo, Japan). The tartrate-resistant acid phosphatase (TRAP) staining kit was purchased from Cell Garage (Tokyo, Japan). Mounting medium (Vecta-Shield) was from Vector Laboratories (Burlingame, CA, USA). Bone Resorption Assay Kit 24 was purchased from PG Research (Tokyo, Japan). Fetal bovine serum (FBS) was acquired from HyClone Laboratories (Logan, UT, USA). Penicillin/streptomycin solution was obtained from Sigma-Aldrich (St. Louis, MO, USA). The RNeasy mini kit was purchased from Qiagen (Valencia, CA, USA). The PrimeScript RT reagent kit and SYBR premixed Ex *Taq* were obtained from Takara Bio (Shiga, Japan).

### Cell culture

The RAW264.7 mouse monocyte cell line [18] was obtained commercially (Dainippon Pharmaceutical Co. Ltd., Osaka, Japan). The cells were maintained in DMEM supplemented with 10% (v/v) heat-inactivated FBS, 1% (v/v) penicillin/streptomycin solution at 37°C in a humidified atmosphere of 95% air and 5% CO_2_. Cells were plated at a density of 1.25×10^4^ cells/cm^2^ and grown in DMEM containing 50 ng/ml RANKL (differentiation medium) for up to 7 days to differentiate osteoclasts, and then the medium was changed every 3 days.

Bone marrow cells from the femurs and tibias of 6–10-week-old male C57Bl/6 mice were used to prepare osteoclasts as described previously [46], [47]. After isolation, cells were suspended in α-MEM supplemented with FBS (10%) and antibiotics (1%) and cultured in T75 tissue culture flasks (15×10^6^ cells per flask) with recombinant human macrophage colony-stimulating factor (50 ng/ml). After 24 h, non-adherent cells were removed and resuspended in α-MEM containing FBS (10%), antibiotics (1%), macrophage colony-stimulating factor (50 ng/ml), and RANKL (50 ng/ml) and plated at 10×10^4^ cells/cm^2^ and cultured in for up to 7 days to differentiate osteoclasts, and then the medium was changed every 3 days. All experiments were conducted in accordance with the guidelines for studies with laboratory animals of Nihon University Experimental Animal Committee.

### TRAP staining

RAW264.7 cells were plated onto 96-well microplates and cultured in differentiation medium with 0 (control), 10^−5^, 10^−4^, or 10^−3^ M nicotine for up to 7 days and then stained using the TRAP staining kit on days 3, 5, and 7 of culture. The fields were moved one by one so that all of the fields were on the bottom of the wells. The number of TRAP-positive multinuclear osteoclasts (more than three nuclei) per well were counted using DIAPHOT phase-contrast microscopy (Nikon, Tokyo, Japan). Cells were visualized using a microscope with a Plan ×10 DL objective (Nikon). Retraction was quantified by tracing the perimeter of each osteoclast to obtain the mean planar cell area under each condition using NIS-Elements D 3.2 software (Nikon).

### Real-time polymerase chain reaction (real-time PCR)

RAW264.7 cells were plated into 6-well microplates and cultured with differentiation medium for up to 7 days. Total RNA was isolated from the cultured cells on days 3, 5, and 7 of culture using an RNeasy Mini Kit (Qiagen). The amount of RNA was measured using NanoDrop 1000 (ND-1000; Thermo Fisher Scientific, Wilmington, DE, USA). Complementary DNA (cDNA) was synthesized from 0.5 µg of DNase-treated total RNA using the PrimeScript RT reagent kit, and the resultant cDNA was subjected to real-time PCR using the SYBR Green I dye. The reactions were performed in a 25 µl total volume containing 12.5 µl SYBR premixed Ex *Taq* (Takara Bio), 0.5 µl (20 mM) each sense and antisense primers (Table 1), 9.5 µl dH_2_O, and 0.5 µg/2 µl cDNA. The PCR assays were performed on a Smart Cycler (Cepheid, Sunnyvale, CA, USA) and analyzed using the Smart Cycler software. The PCR protocol consisted of 35 cycles at 95°C for 5 s and 60°C for 20 s. All real-time PCR experiments were performed in triplicate, and the specificity of the PCR products was verified with melting curve analysis. Calculated values for gene expression levels were normalized to the levels of glyceraldehyde 3-phosphate dehydrogenase (GAPDH) mRNA at the same time point based on our previous studies [48], [49].

**Table 1:** PCR primers used in the experiments.

Target	Primers	GenBank Acc.
α1	5′-GCATGCCTGGCGTGATCTAA-3′	NM_007389.4
	5′-GGGCCTGAACTCCAAACATGA-3′	
α2	5′-GGTCCCAGACGCTAACAGCAA-3′	NM_144803.2
	5′-GGTAAGGCCTCCGACAAGCA-3′	
α3	5′-AGCATTGCACGGTAGGTTCACA-3′	NM_145129.2
	5′-GCTCTGACAACCGAGGCACA-3′	
α4	5′-TACGTGGCTCCAACCACAAGAA-3′	NM_015730.5
	5′-TGTCAGGAGCATCCCAGCAG-3′	
α5	5′-TTCGTCCTGTGGAACACCTGAG-3′	NM_176844.4
	5′-CAACCAGACGTTGGTGGTCATTAG-3′	
α6	5′-GAGCACTCGTCCGATGTTGAAG-3′	NM_021369.2
	5′-AAGACTCTGTCCACCACCATAGCC-3′	
α7	5′-AACCATGCGCCGTAGGACA-3′	NM_007390.3
	5′-CTCAGCCACAAGCAGCATGAA-3′	
α9	5′-AGACCAGTATGACGGGCTGGAC-3′	NM_001081104.1
	5′-GTGTTCACCGGCTCTGAAGACTC-3′	
α10	5′-GAAGGTGTCTCTGGGCGTCA-3′	NM_001081424.1
	5′-GGGCCACAGTAATGCAGATTCA-3′	
CA II	5′-CATTACTGTCAGCAGCGAGCA-3′	NM_009801.4
	5′-GACGCCAGTTGTCCACCATC-3′	
MMP-9	5′-GCCCTGGAACTCACACGACA-3′	NM_013599.2
	5′-TTGGAAACTCACACGCCAGAAG-3′	
cathepsin K	5′-CAGCAGAACGGAGGCATTGA-3′	NM_007802.3
	5′-CCTTTGCCGTGGCGTTATAC-3′	
V-ATPase d2	5′-CCACTGGAAGCCCAGTAAACAGA-3′	NM_175406.3
	5′-GAACGTATGAGGCCAGTGAGCA-3′	
GAPDH	5′-AAATGGTGAAGGTCGGTGTG-3′	NM_008084.2
	5′-TGAAGGGGTCGTTGATGG-3′	

### Sodium dodecyl sulfate-polyacrylamide gel electrophoresis (SDS-PAGE) and Western blotting

After nicotine treatment, cells were cultured in differentiation medium without FBS and nicotine for an additional 24 h. The cells were collected and subjected to SDS-PAGE on 10% polyacrylamide gels (8.3 cm×6.5 cm×0.75 mm) using a discontinuous Tris-glycine buffer system. Medium samples containing 20 µg of extracellular protein were dissolved in 10 µl of sample buffer containing 1% SDS, 2 M urea, 15 mg/ml dithiothreitol, and bromophenol blue, and then heated at 95°C for 5 min before loading onto the gel. Gels were run at 150 V for 60 min. Gel-separated proteins were transferred to a membrane using a semidry electrotransfer unit with a continuous buffer system consisting 39 mM glycine, 48 mM Tris, 0.0375% SDS, and 20% (v/v) methanol at a constant amperage of 0.8 mA/cm^2^ for 60–90 min. On completion of the transfer, the transfer membrane was treated with 25% (v/v) blocking reagent in Tris-buffered saline (TBS) (10 mM Tris, 145 mM NaCl, pH 7.4) at 4°C for 18 h. The sheet was washed in TBS containing Tween 20 (TBS-Tween) and then incubated at room temperature for 90 min with rabbit or goat biotin-labeled polyclonal IgG antibodies against CA II, MMP-9, cathepsin K, V-ATPase, and β-actin (all from Santa Cruz Biotechnology, Santa Cruz, CA, USA). Primary antibodies were diluted 1∶200 in distilled water containing 10% (v/v) blocking reagent. β-actin was used as an internal standard. The membranes were washed in TBS-Tween and incubated at room temperature for 60 min with the appropriate biotin-conjugated secondary antibodies that were diluted 1∶10,000 in distilled water containing 10% blocking agent. The membranes were then washed in TBS-Tween and phosphate-buffered saline (PBS; Nissui Pharmaceutical Co. Ltd., Tokyo, Japan) and then incubated for 30 min at room temperature with horseradish peroxidase-conjugated streptavidin diluted in PBS. Immunoreactive proteins were visualized using a commercial chemiluminescence kit (Amersham Life Sciences, Buckinghamshire, UK) and autoradiography with X-ray film (Eastman Kodak, New Haven, CT, USA). As a control, membranes were exposed to normal rabbit serum; at the same dilution as the primary antibodies. Pre-stained molecular weight standards were run on the same gel.

### Assessment of osteoclast morphology

RAW264.7 cells were plated onto 12-mm coverslips. The cells were treated with 0 (control) or 10^−3^ M nicotine in differentiation medium for 7 days and fixed with 4% (v/v) paraformaldehyde/2% sucrose for 10 min at room temperature. The cells were washed twice with PBS and then permeabilized with 0.1% Triton X-100 in PBS for 10 min at room temperature. Following two 5-min washes with PBS, the cells were incubated in 1% bovine serum albumin for 30 min at room temperature and then stained for filamentous actin using 66 nM Alexa Fluor 488-phalloidin for 20 min at room temperature. The cells were washed twice with PBS and visualized under a fluorescence microscope (BZ 9000; Keyence, Osaka, Japan; Plan Apo λ 20× NA0.75 WD1.00 and Plan Apo λ 4× NA0.20 WD20.00; Nikon).

### Mineral resorption activity and pit formation assay

The mineral resorption assay was performed using the commercially available Bone Resorption Assay Kit 24 (Cosmo Bio Co., Ltd., Tokyo, Japan). RAW264.7 cells were plated onto the assay plate and then cultured in differentiation medium containing phenol red-free DMEM/F-12 instead of DMEM. After 7 days of culture, conditioned media were collected and incubated with resorption assay buffer in a 96-well plate. The fluorescence intensity was measured at an excitation wavelength of 485 nm and an emission wavelength of 535 nm. The cells were washed in 5% NaClO to remove cells, and then the resorbed areas on the plate were visualized under light microscopy.

Osteoclasts were differenciated from RAW264.7 cells or bone marrow cells. Cells were plated on dentin slices (3 mm diameter, 28.3 mm^2^, Wako, Osaka, Japan), and cultured for up to 7 days in each differentiation medium. The cells were removed from the dentin slices, which were stained with Mayer's hematoxylin (Wako) to identify resorption pits. Cells were visualized using a microscope with a Plan ×10 DL objective (Nikon). Pits were quantified by tracing the perimeter of each osteoclast to obtain the mean planar cell area under each condition using NIS-Elements D 3.2 software (Nikon).

### Statistical analysis

All experiments were performed in triplicate or quintuplicate, n = 3 independent experiments. Each value represents the mean±standard deviation (S.D.). The significance of differences was determined using one-way analysis of variance by Bonferroni's multiple comparisons test or Student's t-test. Differences were accepted as statistically significant at *p*<0.05.
